# NECKCHECK project: development and validation of a digital checklist for radiologic assessment of oral cavity squamous cell carcinoma

**DOI:** 10.1007/s00784-026-06934-4

**Published:** 2026-05-22

**Authors:** Sara Maria Ferrero-Coloma, Jose Antonio Quesada, Julián Izquierdo-Luzón, Carlos Ferrero-Coloma, Manuela Sancho-Mestre, Elena Garcia-Garrigós, Avelino Pereira-Expósito, Vicente Gil-Guillen

**Affiliations:** 1 Department of Otolaryngology, Dr. Balmis General University Hospital of Alicante, Alicante, Spain; 2 Department of Clinical Medicine, Miguel Hernández University, San Juan de Alicante, Spain; 3 Department of Otolaryngology, General University Hospital of Elda, Elda, Spain; 4 Department of Anesthesiology, Dr. Balmis General University Hospital of Alicante, Alicante, Spain; 5 Department of Radiology, Dr. Balmis General University Hospital of Alicante, Alicante, Spain; 6 Medical Research Department, General University Hospital of Elda, Elda, Spain; 7 Departamento Medicina Clinica de la UMH, Crta. Nacional 332 s/n, 03550 San Juan de Alicante, Spain

**Keywords:** Oral cavity squamous cell carcinoma, Diagnostic imaging, Interobserver agreement, Computed tomography, Magnetic resonance imaging, Radiologic checklist

## Abstract

**Objectives:**

Oral cavity squamous cell carcinoma (OCSCC) is one of the most common malignancies affecting the oral region and requires accurate imaging assessment for staging and treatment planning. However, variability in radiologic interpretation may lead to inconsistencies in clinical decision-making. The aim of this study was to develop and evaluate a structured radiologic checklist (NECKCHECK) to improve diagnostic accuracy and interobserver agreement in the imaging assessment of OCSCC.

**Materials and methods:**

NECKCHECK was developed following a comprehensive narrative review of the literature focusing on relevant staging features of OCSCC using computed tomography (CT) and magnetic resonance imaging (MRI). Key radiologic parameters were organized into a digital checklist designed to guide systematic image interpretation. A concordance study was performed in which otolaryngologists independently evaluated anonymized CT and MRI examinations of patients with OCSCC, both with and without checklist support. Expert radiologist assessment served as the reference standard. Interobserver agreement was assessed using Cohen’s kappa coefficient, and diagnostic accuracy was measured as the proportion of correct responses.

**Results:**

Use of the checklist significantly improved interobserver agreement, with mean Cohen’s kappa increasing from 0.44 to 0.72. Diagnostic accuracy also improved significantly, with correct responses increasing from 67.1% to 84.7% (*p* < 0.001). Perfect agreement was achieved for key parameters such as tumor size and depth of invasion.

**Conclusions:**

Implementation of a structured radiologic checklist improves both diagnostic accuracy and interobserver agreement in the imaging evaluation of OCSCC.

**Clinical relevance:**

NECKCHECK may support more standardized radiologic assessment of oral cavity squamous cell carcinoma and improve multidisciplinary clinical decision-making in oral cancer management.

**Supplementary Information:**

The online version contains supplementary material available at https://doi.org/10.1007/s00784-026-06934-4.

## Introduction

According to the World Health Organization (WHO), it is estimated that more than 350,000 new cases of oral cavity cancer are diagnosed worldwide each year, with approximately 177,000 deaths attributable to this disease [1]. The global epidemiology of oral cavity squamous cell carcinoma (OCSCC) has undergone significant changes inrecent years, with a consistent increase in prevalence observed among women compared with men. These changes are primarily attributed to shifts in lifestyle and behavioural patterns, as well as to increased societal awareness and the implementation of national preventive and early detection programs worldwide [2].

Radiologic studies play a central role in the management of patients with oral cavity tumors. The integration of clinical and radiologic assessment enables a more precise evaluation of tumor extent and supports appropriate treatment planning. Owing to the complex anatomy of the oral cavity and its adjacent structures, imaging plays a fundamental role not only in locoregional staging but also in the assessment of distant metastatic disease and post-treatment surveillance [3]. The appropriate selection and use of imaging modalities are therefore critical for the diagnosis, management and prognosis of patients with OCSCC [4] The interpretation of radiologic studies is commonly performed by medical professionals in clinical practice, given the importance of obtaining accurate information that may directly influence patient management and treatment decisions [5]. In addition, diagnostic workflows and management strategies for OCSCC may vary across healthcare systems and clinical settings, reflecting differences in specialist roles and organizational structures. Medical specialty residency programs, such as those in Otolaryngology, Head and Neck Surgery (OHNS), require structured and standardized training frameworks to strengthen radiologic education, with the objective of optimizing patient care and promoting effective interdisciplinary collaboration between otolaryngologists and radiologists. However, these educational frameworks are not universally established nor consistently implemented, raising concerns regarding potential gaps in radiologic training among specialists [6].

In this context, the systematic review and standardization of key radiologic principles applied to OCSCC may lead to improved diagnostic effectiveness. It is hypothesized that the development of a novel diagnostic tool, grounded in published and digitized scientific evidence, enhances the accuracy, validity, and overall performance of image interpretation and, consequently, reduces errors associated with the radiologic assessment of OCSCC by physicians who are not radiology specialists, such as otolaryngologists. The primary objective of this study is to determine whether a computerized decision-support tool, based on a narrative review of the scientific literature, improves diagnostic reliability in the interpretation of radiologic images of patients with OCSCC when used by otolaryngology specialists. Secondary objectives include assessing whether this digital tool improves diagnostic validity and clinical utility, as measured by the number of correct interpretations, as well as identifying epidemiologic and professional factors related to both participating clinicians and the study population, together with tool-specific variables, that are associated with higher levels of diagnostic agreement or with discrepancies between otolaryngologists and radiologists, considered the reference standard. Finally, the integration of the developed tool into an expanded digital platform is proposed to facilitate broader dissemination and use, enhancing accessibility in both clinical and educational settings.

## Materials and methods

This investigation was designed as a three-stage sequential study comprising: (1) a structured narrative literature review to identify key radiologic features relevant to image interpretation, staging, and treatment planning in OCSCC; (2) development and digital implementation of a standardized imaging checklist based on the synthesized evidence; and (3) an observational concordance study assessing agreement in radiologic image interpretation between radiologists and otorhinolaryngologists (ENTs).

The study was conducted in accordance with the ethical principles of the Declaration of Helsinki and was approved by the Ethics Committee of the General University Hospital of Elda (protocol 2022/55PI). The project was developed within the framework of a doctoral research program.

### Stage 1: Literature review and evidence synthesis

A structured literature search was performed in PubMed, Google Scholar, and the Cochrane Library for studies published between January 2005 and January 2024, with the final search conducted in January 2024. The search strategy incorporated MeSH terms including “Oral Cavity,” “Carcinoma”, “Computed Tomography” and “Magnetic Resonance Imaging”. Predefined inclusion and exclusion criteria were applied. Eligible studies included systematic reviews, meta-analyses, and observational studies evaluating computed tomography (CT) and/or magnetic resonance imaging (MRI) in oral cavity carcinoma and reporting imaging findings relevant to tumor extension, staging, or treatment planning. Non–peer-reviewed publications, case reports, articles not available in English or Spanish, and studies lacking sufficient imaging data were excluded.

Methodological quality was appraised according to study design, relevance to imaging evaluation in OCSCC, clarity of radiologic outcome definitions, and consistency with established staging and treatment guidelines. Articles were reviewed in three consecutive rounds by a multidisciplinary team composed of otorhinolaryngologists specialized in head and neck oncology and radiologists with expertise in head and neck imaging. Imaging-related concepts aligned with international staging recommendations and the TNM classification system were identified and selected by consensus. Priority was given to high-quality systematic reviews and meta-analyses, although relevant observational studies, reference textbooks, and clinical practice guidelines were also considered.

### Stage 2: Checklist development and digital implementation

Based on the synthesized evidence from the literature and multidisciplinary agreement, key imaging domains were operationalized into a standardized checklist structured around primary tumor location and anatomical subsite, tumor extent and depth of invasion, involvement of adjacent anatomical structures, nodal disease characteristics, and additional findings relevant to TNM staging and treatment planning. The checklist was implemented as an open-access web-based tool developed using the GoodBarber platform and is accessible at https://www.neckcheck.es (Supplementary material 1.1). The interface guides users sequentially through predefined imaging domains and generates a structured summary upon completion. No patient-identifiable data are stored or processed by the application, ensuring compliance with data protection standards. In addition, illustrative imaging examples were developed to support selected checklist items and facilitate conceptual clarification (Supplementary material 1.2). These visual aids were incorporated into the digital interface as explanatory material and were not part of the image datasets used for the concordance analysis.

### Stage 3: Observational concordance study

 Patients diagnosed with OCSCC between 2010 and 2024 constituted the unit of analysis. All patients were managed according to established clinical practice guidelines within hospitals of the Spanish National Health System. Inclusion criteria required complete, diagnostic-quality CT and/or MRI examinations accompanied by an official radiology report issued by a board-certified radiologist. Patients were excluded if imaging datasets were incomplete, lacked a formal report, or demonstrated tumor extension involving multiple anatomical regions that precluded isolated oral cavity assessment. The reference standard was established by radiologists dedicated to head and neck imaging, with several years of experience in this subspecialty. To ensure consistency and reduce variability, the number of radiologists involved in this process was intentionally limited.

Eligible imaging studies were retrospectively retrieved from institutional Picture Archiving and Communication System (PACS) repositories. CT examinations were performed using multidetector scanners with standardized acquisition parameters, including 2-mm slice thickness with 1-mm overlap. Intravenous iodinated contrast was administered, and image acquisition was performed approximately 70 s after contrast injection. MRI protocols included T1-weighted sequences acquired with and without intravenous contrast, T2-weighted sequences for soft tissue characterization, and fat-suppressed sequences. Protocols were harmonized to minimize motion- and metal-related artifacts and complied with quality standards recommended by the European Society of Radiology.

Actively practicing otorhinolaryngologists and senior residents with at least three completed years of training from public hospitals were recruited as evaluators, whereas retired or non–clinically active physicians were excluded. Participants independently reviewed anonymized imaging datasets under blinded and randomized conditions. Limited demographic information, including age and sex, was provided in anonymized form and linked to each case through a unique identification code.

Image assessment was conducted in two sequential phases. In the first phase, participants reviewed three OCSCC cases without structured decision support, providing a free-text interpretation and assigning TNM staging. After a 7-day washout period to reduce recall bias, a second phase was performed using three different OCSCC cases following the same evaluation procedure but with access to the digital checklist via QR code on personal smartphones; compatible devices were provided when necessary. Each participant evaluated six distinct cases in total, and no feedback was provided between phases. Image interpretation was performed on calibrated 27-inch diagnostic monitors (2560 × 1440 pixels) under standardized ambient lighting conditions, and descriptive data from both participants and imaging cases were systematically recorded.

Statistical analyses were performed using SPSS (version 28) and R (version 4.3.1). Categorical variables were expressed as frequencies and percentages, and continuous variables as means and standard deviations. Data distribution was assessed using the Kolmogorov–Smirnov test. Group comparability was evaluated using chi-square tests for categorical variables and Student’s t tests for continuous variables. Agreement between ENT specialists and radiologists was analyzed for each checklist item as a binary outcome (concordant vs. non-concordant), and accuracy in TNM T and N classification was assessed. Cohen’s kappa coefficients with 95% confidence intervals were calculated for each item. Overall concordance was estimated using a meta-analytic approach treating individual checklist items as units of analysis and kappa values as effect sizes. A mixed-effects model was fitted with professional group as a moderator, and forest plots were generated. Additionally, proportions of correct responses were compared using chi-square tests, and the total number of correct responses per participant was compared between phases using Student’s t test.

## Results

### Review results

**Fig. 1:**
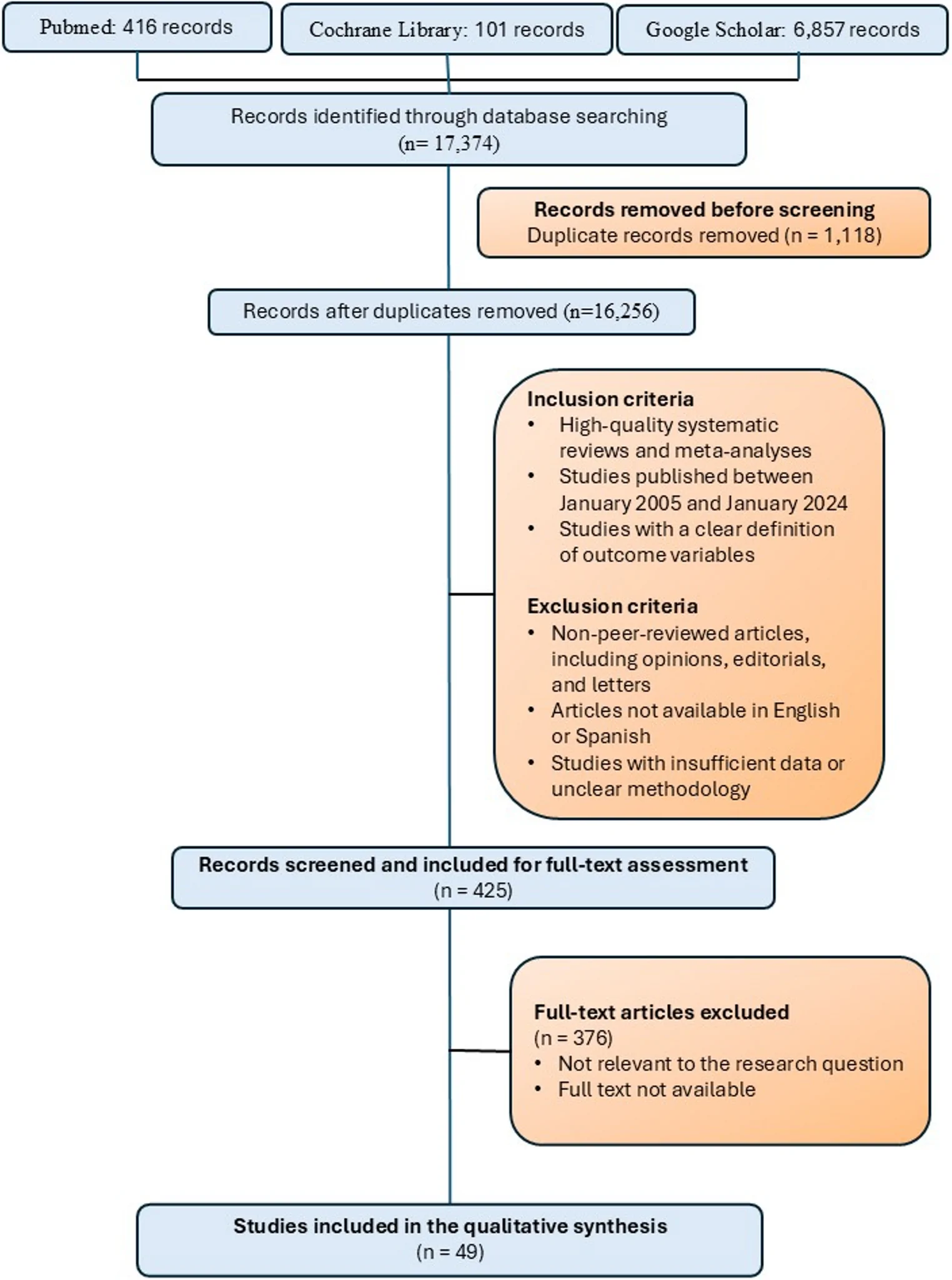
Literature search and study selection flow diagram

The literature selection process is summarized in Fig. 1. The database search yielded 17,374 records, which were reduced to 16,256 unique articles after removal of duplicates. After sequential screening and full-text evaluation based on predefined eligibility criteria, 49 studies were retained for qualitative synthesis. These studies formed the methodological foundation for the identification and consolidation of the imaging features included in the final checklist.

### Key imaging elements for oral cavity squamous cell carcinoma: framework for checklist development

Following the literature synthesis, a set of core imaging elements essential for a comprehensive and reproducible assessment of OCSCC was defined (Table 1). These elements were selected based on their relevance for TNM staging, prognostic stratification, and surgical decision-making. To promote systematic evaluation and reduce interpretative variability, all checklist items were designed to be completed using dichotomous or structured multiple-choice responses. The checklist guides the user through sequential tumour assessment, starting with primary tumour characterization and progressing to locoregional extension.

**Table 1:** Key radiologic features in oral cavity squamous cell carcinoma

Item	Imaging modality	Key imaging findings	Surgical implications	TNM classification [7]
Tumor size	MRI, CT	Essential prognostic parameter	Associated with local recurrence and survival in oral cavity cancer	≤ 2 cm: T1 or T2 (depending on DOI) >2 cm and ≤ 4 cm: T2 or T3 (depending on DOI) >4 cm: T3
Depth of invasion (DOI)	MRI	Perpendicular measurement to the deepest point of infiltration	Increasing DOI is associated with higher risk of regional metastases and the need for elective neck dissection [8]	DOI ≤ 10 mm: T2 DOI > 10 mm: T3
Extrinsic tongue musculature involvement	MRI	From medial to lateral: genioglossus, hyoglossus, styloglossus, and mylohyoid muscles	Predicts the likelihood of requiring neck dissection [9]	Hyoglossus or styloglossus involvement implies DOI > 4 mm → T3
Lingual septum crossing	MRI (preferred); T1-weighted post-contrast and T2 sequences	Best assessed on coronal planes; midline should be identified	Precludes partial glossectomy and hemiglossectomy; reconstruction should be considered [10]	Likely T2–T3 depending on tumor size
Fascial spread along the pterygomandibular raphe (PMR)	MRI (preferred): T1-weighted post-contrast and T2; MDCT	Location inferred by identifying the junction between the superior pharyngeal constrictor and buccinator muscle	Invasion is often difficult to resect and associated with high local recurrence rates [11]	May involve the medial pterygoid muscle → T4b.
Base of tongue extension	MRI	Typically associated with posteriorly located tumors	Requires wider resections, sometimes combined with TOUS or TORS	
Bone erosion	MDCT (best for cortical bone); MRI (best for medullary bone); slice thickness 1 mm [12]	Evaluate mandible, maxilla, hard palate, pterygoid processes, and skull base; cortical interruption on CT or diffuse hypointensity on MRI	Bone erosion or cortical interruption on CT, or hypointense margins on MRI across all sequences [12].	Superficial: T3 Deep invasion: T4a Skull base: T4b
Sublingual space involvement	MRI (T1); MDCT	Low attenuation similar to fat on CT	Extended resection up to the mylohyoid muscle	Probable T3.- T4a
Masticator space involvement	Contrast-enhanced MDCT; MRI (T1 post-contrast and T2)	Identification of medial and lateral pterygoid muscles, masseter muscle, and mandibular ramus	Traditionally considered a criterion of unresectability [13]	T4b
Submandibular gland involvement	MRI (T1); MDCT (for sialolithiasis or when MRI is contraindicated)	Identification of the anterior belly of the digastric muscles, mylohyoid muscle, and hyoid bone	Higher probability of submandibular gland involvement in ≥N2 disease, extracapsular spread, or direct invasion by advanced primary tumors	Likely T3–T4
Internal carotid artery (ICA) involvement	MRI (preferred); T1-weighted post-contrast and T2 sequences	Carotid encasement if involvement > 270°, associated with very poor prognosis [14]	If surgery is performed, arterial resection is required	T4b
Perineural spread (PNS)	MRI (preferred); high-resolution isotropic volumetric T1-weighted sequences, with or without fat suppression	Nerve enhancement and thickening, obliteration of fat planes, foraminal enlargement, and denervation atrophy [15]	Predictor of cervical lymph node involvement [16]	

The key imaging elements considered in the checklist are outlined below:

- Tumor size: Tumor size is a fundamental parameter in TNM classification for OCSCC and is closely related to local control and survival. Lesions ≤ 2 cm may correspond to T1 or T2 depending on depth of invasion (DOI), tumors > 2 cm and ≤ 4 cm to T2 or T3, and those > 4 cm to T3 or T4, also conditioned by DOI.
- DOI: It is measured by drawing a reference line between the adjacent normal mucosal surfaces and a perpendicular line to the deepest point of tumor infiltration. Increasing DOI is associated with a higher risk of regional nodal metastases and the need for elective neck dissection. Radiological DOI correlates with pathological DOI but may overestimate it by 2–3 mm. Lesions not detectable on MRI typically correspond to a pathological DOI < 4 mm [8].
- Extrinsic tongue musculature involvement: Assessment includes the genioglossus, hyoglossus, styloglossus, palatoglossus, and the mylohyoid muscle of the floor of the mouth. MRI evidence of invasion of the styloglossus or hyoglossus muscles correlates with DOI > 4 mm and has implications for prognosis and elective neck management [8, 9].
- Lingual septum crossing: Midline involvement is defined when the primary tumor reaches or crosses the lingual septum. This finding is strongly associated with contralateral cervical nodal metastases and adversely impacts disease-free and overall survival, influencing both surgical planning and neck management strategies [10, 17].
- Fascial spread along the pterygomandibular raphe: Invasion of the pterygomandibular raphe is a critical determinant of surgical approach and is associated with a high risk of local recurrence [11]. MRI, particularly axial T2- and T1-weighted sequences, is useful for assessing soft tissue and fat plane involvement in this region [18].
- Base of tongue extension: Posteriorly located OCSCCs may extend into the tongue base. Surgical resection in this area may compromise lingual neurovascular structures, with potential swallowing, respiratory, and phonatory consequences.
- Bone erosion: Cortical interruption on CT or diffuse hypointensity on MRI across all sequences indicates osseous invasion [12]. CT is superior for detecting subtle cortical involvement, whereas MRI is more sensitive for medullary invasion. Bone erosion represents an adverse prognostic factor and may complicate both diagnosis and treatment [19–21].
- Maxillary sinus, pterygoid processes, and skull base involvement: Involvement of these regions is uncommon but clinically significant. Multidetector CT is typically the first-line modality. Maxillary sinus invasion classifies tumors as T4a and may be associated with palatal nerve involvement and posterior skull base extension [21].
- Sublingual space involvement: This space is predominantly fatty and communicates across the midline beneath the lingual frenulum. Identification of the mylohyoid and hyoglossus muscles is essential for detecting pathological extension [22, 23].
- Submandibular gland involvement: Although direct submandibular gland invasion is rare, it may occur in advanced tumors or in the presence of high nodal burden with extracapsular spread. These findings influence decisions regarding gland preservation during neck dissection [22, 24, 25].
- Masticator space involvement: Invasion of the masticator space complicates surgical management and is often associated with advanced disease. While traditionally considered unresectable in T4b tumors, selected cases may benefit from surgery in experienced multidisciplinary centers [13, 26].
- Internal carotid artery (ICA) involvement: Tumor encasement exceeding 270° of the ICA circumference on CT or MRI is associated with extremely high mortality [27]. Lesser degrees of contact are less predictive of vascular invasion [14]. Doppler ultrasound may provide complementary hemodynamic information [28].
- Perineural spread (PNS): PNS is associated with reduced survival and increased nodal involvement [29]. MRI is the preferred modality, with primary signs including nerve enhancement and enlargement, obliteration of perineural fat, foraminal widening, and intracranial extension [15]. Secondary signs include denervation atrophy of the masticatory muscles or tongue [16].

After completion of all items, the user assigns the final T classification using a structured reference table linking patterns of involvement to TNM categories. A summarized report of responses is then generated and identified by a unique user-defined code.

### Study concordance results

An image repository was created specifically for this study, consisting of 45 representative cases of OCSCC selected from an initial pool of more than 500 radiological examinations. Case selection was based on predefined inclusion and exclusion criteria and required high-quality visualization of deep anatomical structures, tumor sublocalization, and muscular planes. After blinded randomization, each case was evaluated in two independent rounds, resulting in a total of 90 image interpretations.

A descriptive analysis of baseline characteristics confirmed the comparability of the two evaluation rounds. No statistically significant differences were observed in case origin (Alicante vs. Valencia; *p* = 0.803), sex distribution (33.3% female and 66.7% male in both rounds; *p* = 1.000), or mean age (65.6 (12.8) vs. 64.7 (11.8) years; *p* = 0.704), with age following a normal distribution (Kolmogorov–Smirnov test, *p* = 0.076). Tumor stage distribution (stages II, III, IVa, and IVb) was also comparable (*p* = 0.494), as was the low prevalence of distant metastases (6.7% vs. 15.6%; *p* = 0.180). These findings indicate homogeneity between the two evaluation rounds and support the internal validity of the concordance analysis. (Table 2)

**Table 2:** Comparative descriptive analysis of OCSCC imaging cases

		Without checklist		With checklist		
		*n*	%	*n*	%	*p*-values
**Case origin**	Alicante	35	77.8%	34	75.6%	0.803
	Valencia	10	22.2%	11	24.4%	
**Sex**	Female	15	33.3%	15	33.3%	1,000
	Male	30	66.7%	30	66.7%	
**Stage at diagnosis**	I	1	2.2%	0	0.0%	0.494
	II	8	17.8%	6	13.3%	
	III	12	26.7%	8	17.8%	
	IVa	16	35.6%	18	40.0%	
	IVb	8	17.8%	13	28.9%	
**Distant metastasis (M)**	0	42	93.3%	38	84.4%	0.180
	1	3	6.7%	7	15.6%	
**Age (years)**	Media (SD)	65.6	(12.8)	64.7	(11.8)	0.704

All image sets were independently assessed by 15 otorhinolaryngologists selected according to predefined eligibility criteria, ensuring a consistent level of clinical training. Item-level analysis (Table 3) demonstrated a clear improvement in diagnostic performance with the use of the structured checklist. The mean number of correct responses increased from 11.4 ± 2.5 without checklist support to 14.4 ± 1.5 with checklist use, out of a maximum of 17 items (67.1% vs. 84.7%; *p* < 0.001). This improvement was accompanied by a reduction in response dispersion, reflected by a lower standard deviation in the checklist-assisted evaluations.

**Table 3:** Item-level analysis of correct responses by OCSCC group

		Without checklist		Checklist		
		*n*	%	*n*	%	*p*-values
**Tumor sublocalization**	Incorrect	20	71.4%	8	28.6%	0.006
	Correct	25	40.3%	37	59.7%	
**Tumor size**	Incorrect	30	100.0%	0	0.0%	< 0.001
	Correct	15	25.0%	45	75.0%	
**DOI**	Incorrect	39	95.1%	2	4.9%	< 0.001
	Correct	6	12.2%	43	87.8%	
**Extrinsic tongue musculature involvement**	Incorrect	19	65.5%	10	34.5%	0.042
	Correct	26	42.6%	35	57.4%	
**Lingual septum crossing**	Incorrect	11	68.8%	5	31.3%	0.098
	Correct	34	45.9%	40	54.1%	
**Perineural spread**	Incorrect	10	62.5%	6	37.5%	0.270
	Correct	35	47.3%	39	52.7%	
**Base of tongue extension**	Incorrect	4	50.0%	4	50.0%	1,000
	Correct	41	50.0%	41	50.0%	
**Bone erosion**	Incorrect	10	76.9%	3	23.1%	0.036
	Correct	35	45.5%	42	54.5%	
**Skull base involvement**	Incorrect	1	50.0%	1	50.0%	1,000
	Correct	44	50.0%	44	50.0%	
**Sublingual space involvement**	Incorrect	11	52.4%	10	47.6%	0.803
	Correct	34	49.3%	35	50.7%	
**Submandibular space involvement**	Incorrect	7	50.0%	7	50.0%	1.000
	Correct	38	50.0%	38	50.0%	
**Masticator space involvement**	Incorrect	8	61.5%	5	38.5%	0.368
	Correct	37	48.1%	40	51.9%	
**Internal carotid artery involvement**	Incorrect	2	66.7%	1	33.3%	0.557
	Correct	43	49.4%	44	50.6%	
**Perineural spread**	Incorrect	6	75.0%	2	25.0%	0.138
	Correct	39	47.6%	43	52.4%	
**Cervical lymph node involvement**	Incorrect	16	76.2%	5	23.8%	0.006
	Correct	29	42.0%	40	58.0%	
**T classification**	Incorrect	38	59.4%	26	40.6%	0.005
	Correct	7	26.9%	19	73.1%	
**N classification**	Incorrect	22	47.8%	24	52.2%	0.673
	Correct	23	52.3%	21	47.7%	
Total correct responses (0–17)* Media (SD)	Correct	11.4	(2.5)	14.4	(1.5)	< 0.001

* Normality test for total correct responses: *p* < 0.001

Statistically significant improvements were observed in several clinically relevant parameters, including depth of invasion (12.2% vs. 87.8%; *p* < 0.001), tumor size (25.0% vs. 75.0%; *p* < 0.001), T classification (26.9% vs. 73.1%; *p* = 0.005), and tumor sublocalization (40.3% vs. 59.7%; *p* = 0.006). Additional gains were noted for nodal involvement, muscular invasion, and bone erosion. Other items showed a numerical increase in correct responses without reaching statistical significance, likely due to lower prevalence or higher baseline agreement.

**Fig. 2:**
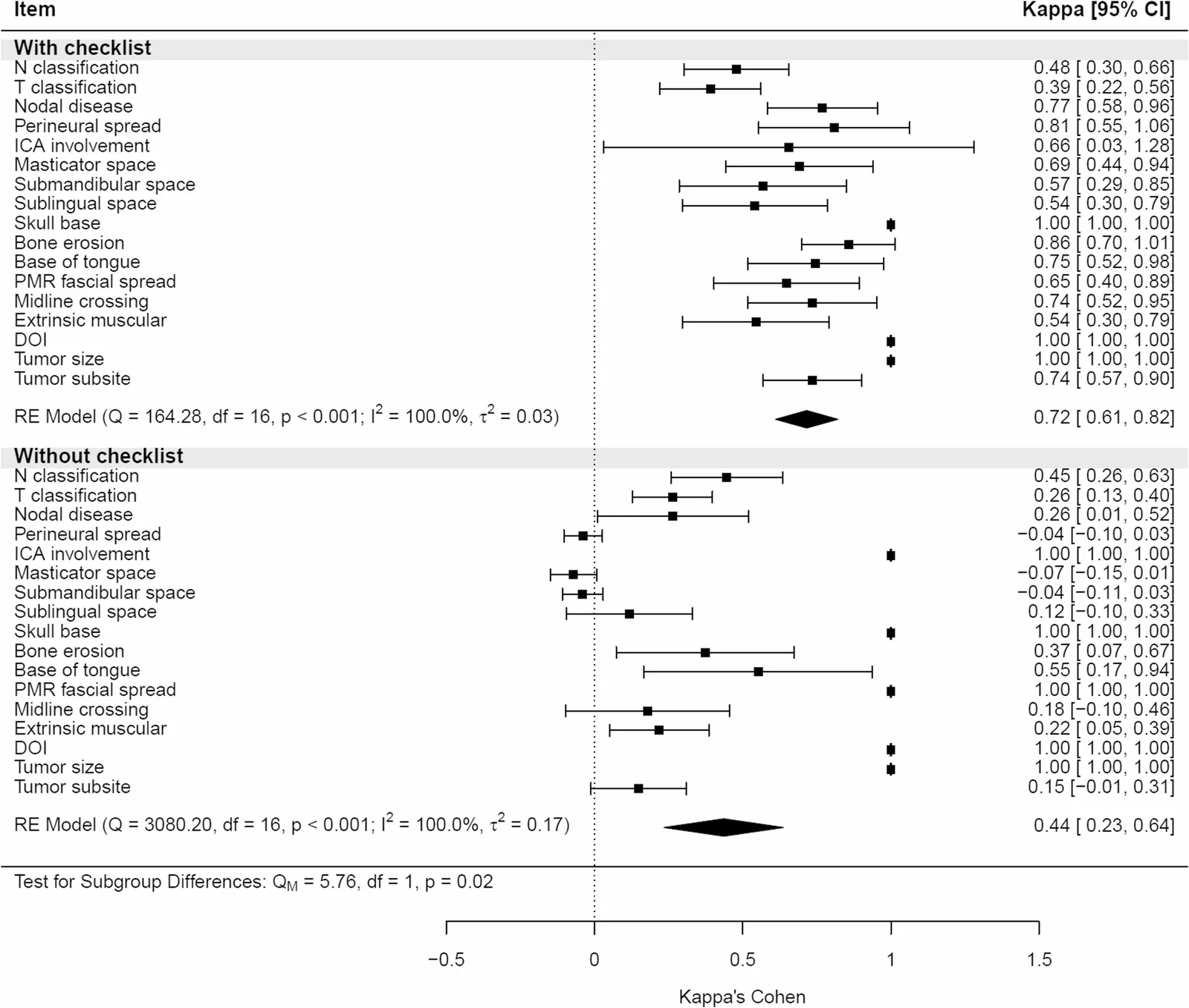
Concordance analysis for each item within each group

Interobserver concordance was assessed using Cohen’s kappa coefficients and visualized with forest plots (Fig. 2). Overall agreement was significantly higher when the checklist was used, with a mean kappa of 0.72 (95% CI: 0.61–0.82), compared with 0.44 (95% CI: 0.23–0.64) without checklist support (*p* < 0.02). Perfect agreement (kappa = 1.00) was achieved for DOI, tumor size, and skull base involvement. In contrast, several complex items showed low or negative agreement in the absence of structured guidance. Although heterogeneity across items was high (I² = 100%), between-item variance was substantially lower in the checklist-assisted analysis (τ² = 0.03 vs. 0.17), indicating greater consistency among evaluators.

Overall, these findings demonstrate that the use of a structured imaging checklist significantly enhances diagnostic accuracy and interobserver concordance in the radiological assessment of OCSCC, particularly for high-impact features relevant to TNM staging and treatment planning.

## Discussion

The results of the present study indicate that the implementation of the NECKCHECK tool is associated with a substantial improvement in diagnostic agreement between otolaryngologists and radiologists in the evaluation of oral cavity squamous cell carcinoma. The kappa index increased from 0.44, reflecting a low level of agreement, to 0.72, a value interpreted as substantial concordance according to the Landis and Koch classification. This increase represents a clinically relevant reduction in interobserver variability and supports the usefulness of the checklist as a complementary aid in the radiological interpretation of OCSCC. The observed improvement in kappa values reflects greater consistency in image-based assessments and highlights the contribution of structured evaluation frameworks in complex oncological imaging.

In parallel with the enhancement in qualitative agreement, the analysis of the total number of correct responses showed a statistically significant increase when the checklist was used. This finding confirms that NECKCHECK not only improves interobserver reliability but also results in higher overall diagnostic accuracy. The most relevant gains were observed in parameters of major clinical importance, including tumor sublocalization, maximum tumor diameter, DOI, T classification, bone erosion, and cervical lymph node involvement. Among these, the improvement in DOI assessment is particularly noteworthy, as DOI is a well-established predictor of cervical lymph node metastasis and a key criterion for indicating elective neck dissection in oral cavity cancer. The accuracy of DOI identification increased from low baseline values to levels approaching 90%, underscoring the clinical relevance of a systematic, checklist-based approach [8]. Interobserver variability in radiologic interpretation is influenced by multiple factors, including differences in training, experience, and familiarity with head and neck imaging. The heterogeneity observed across certain checklist items reflects the inherent complexity of specific anatomical regions and imaging features. In this context, structured tools such as NECKCHECK may help reduce variability by promoting a systematic approach and minimizing the risk of overlooking relevant findings, particularly in more complex or less familiar parameters. (C3.2)

Significant improvements were also observed in the estimation of tumor size and T category, variables that are frequently underestimated in routine clinical practice and that have undergone substantial revisions in recent editions of the AJCC Cancer Staging Manual [7]. The assessment of mandibular or cortical bone erosion improved notably with checklist use, addressing a known diagnostic challenge in both computed tomography and magnetic resonance imaging. Previous studies have reported limited sensitivity of conventional imaging modalities in detecting bone invasion, reinforcing the need for structured interpretative strategies [19, 30]. In this context, NECKCHECK contributes by guiding clinicians through a standardized sequence of key radiological features.

The detection of cervical lymphadenopathy increased significantly, with accuracy rising from 42.0% to 58.0%. This finding is particularly relevant in oral cavity cancer, where nodal involvement is strongly associated with reduced survival and directly influences surgical planning and adjuvant treatment decisions. Radiological factors such as increased DOI, muscular invasion, and midline crossing are recognized predictors of cervical metastasis. The checklist facilitated a more systematic identification of these high-risk features, functioning not only as a standardization tool but also as a cognitive aid that reduces the likelihood of overlooking subtle yet prognostically significant findings. However, it is important to emphasize that structured tools such as NECKCHECK are not intended to replace comprehensive clinical reasoning or multidisciplinary decision-making, but rather to support a systematic and thorough evaluation of radiological findings.

To facilitate clinical implementation, a mobile web-based application, NECKCHECK, was developed and made freely accessible. The digital format provides an interactive and standardized environment for image interpretation, aligning with contemporary clinical workflows and educational practices. Comparable digital platforms have been shown to improve radiological knowledge acquisition and user engagement, and NECKCHECK offers similar advantages by integrating radiological criteria with clinically oriented decision-making in head and neck oncology [31]. The digital and accessible design of NECKCHECK facilitates its potential integration into routine clinical workflows, particularly as a structured aid for radiologic image interpretation. The checklist is primarily intended for clinicians involved in the management of oral cavity cancer, particularly otorhinolaryngologists and other specialists with limited experience in radiologic interpretation. This accessible design supports its potential integration into routine clinical workflows, particularly as a structured aid for radiologic image interpretation. The checklist is primarily intended for clinicians involved in the management of oral cavity cancer, particularly otorhinolaryngologists and other specialists with limited experience in radiologic interpretation.

In addition to its clinical utility, NECKCHECK has considerable potential as an educational resource. Its structured and sequential design supports the learning of complex radiological criteria and may be particularly beneficial for residents and early-career specialists. By promoting systematic image evaluation and minimizing non-protocolized readings, the checklist contributes to more homogeneous training and supports the development of diagnostic competencies in oral cavity cancer imaging.

Certain considerations should be taken into account when interpreting these findings. The number of cases evaluated per participant and the controlled study setting may influence the direct translation of results to routine clinical practice. Furthermore, the checklist was specifically designed to focus on radiological parameters as a structured aid for image interpretation, while clinical information remains an essential component of comprehensive patient assessment. It should also be considered that the roles of different specialists involved in OCSCC management may vary across healthcare systems. In our setting, otorhinolaryngologists play an active role in diagnostic and therapeutic decision-making, although this may differ in other contexts.

From a broader perspective, the increasing emphasis on digital image analysis in oncological diagnosis highlights the relevance of structured tools capable of extracting clinically meaningful information from radiological studies. Checklist-based systems such as NECKCHECK provide a suitable framework for future integration with artificial intelligence applications, as they define explicit variables and decision points that are amenable to supervised learning approaches. Although current AI models have not yet reached widespread clinical applicability in this field, structured diagnostic tools represent an important step toward hybrid systems combining automated analysis with expert clinical interpretation. Future directions include multicenter validation, evaluation in real-world clinical settings, and potential expansion of the tool to other areas within head and neck oncology, as well as its assessment in different specialist groups.

## Conclusion

In conclusion, the use of the NECKCHECK checklist is associated with improved interobserver agreement and diagnostic accuracy in the radiological assessment of OCSCC. The tool enhances the identification of key parameters relevant for TNM staging and therapeutic planning while reducing diagnostic variability. NECKCHECK should be regarded as a complementary and educational resource that supports standardized image interpretation within multidisciplinary clinical practice. However, it should be emphasized that this tool is intended as a complementary resource and should not replace comprehensive clinical assessment or multidisciplinary decision-making.

## Supplementary Information

Below is the link to the electronic supplementary material.

Supplementary Material 1

## Data Availability

The datasets generated and analyzed during the current study are not publicly available due to ethical and privacy restrictions related to patient imaging data but are available from the corresponding author on reasonable request.
