# Supplementary material for: NECKCHECK project: development and validation of a digital checklist for radiologic assessment of oral cavity squamous cell carcinoma
Source: Clin Oral Investig. 2026 May 22;30(6):243. doi: 10.1007/s00784-026-06934-4 (PMC13194212; doi:10.1007/s00784-026-06934-4)
Supplement: Supplementary file 1 — Supplementary Material 1 [file 784_2026_6934_MOESM1_ESM.docx]

**Supplementary material 1.1**

Web application


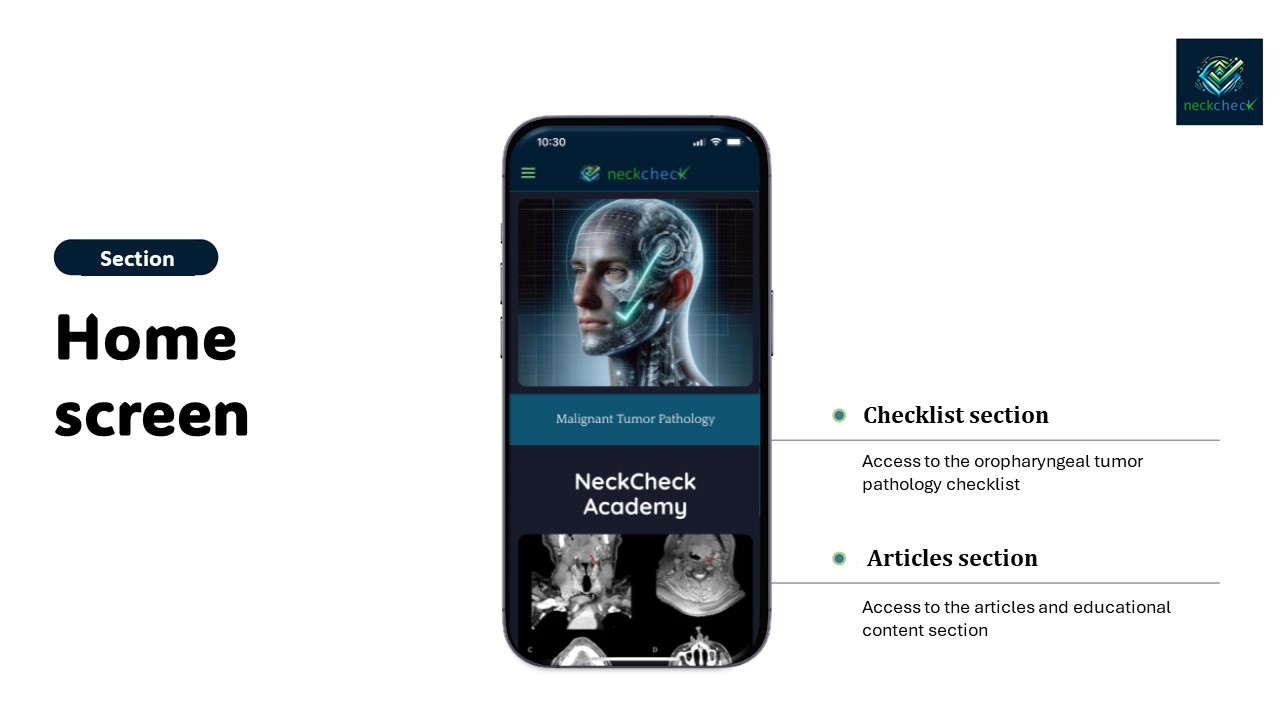


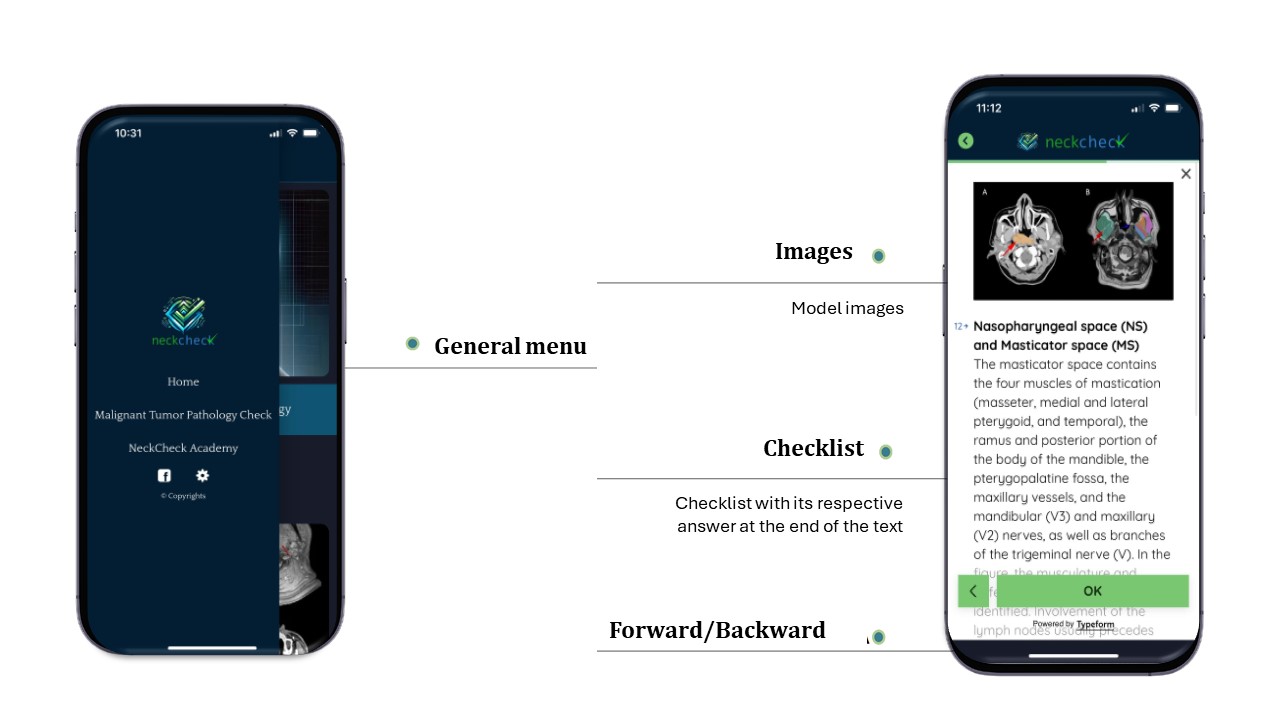


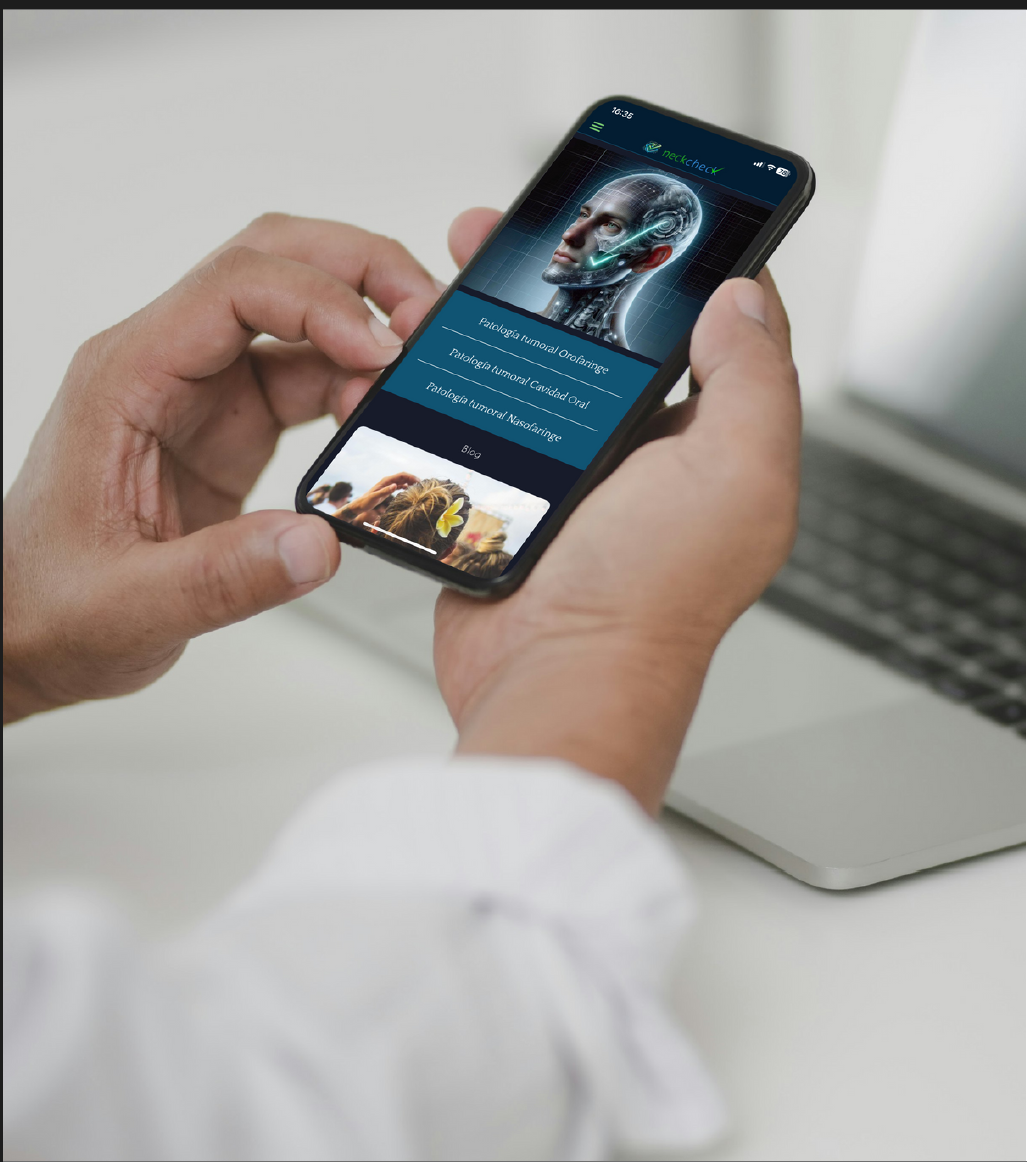


**Supplementary material 1.2**

Model images


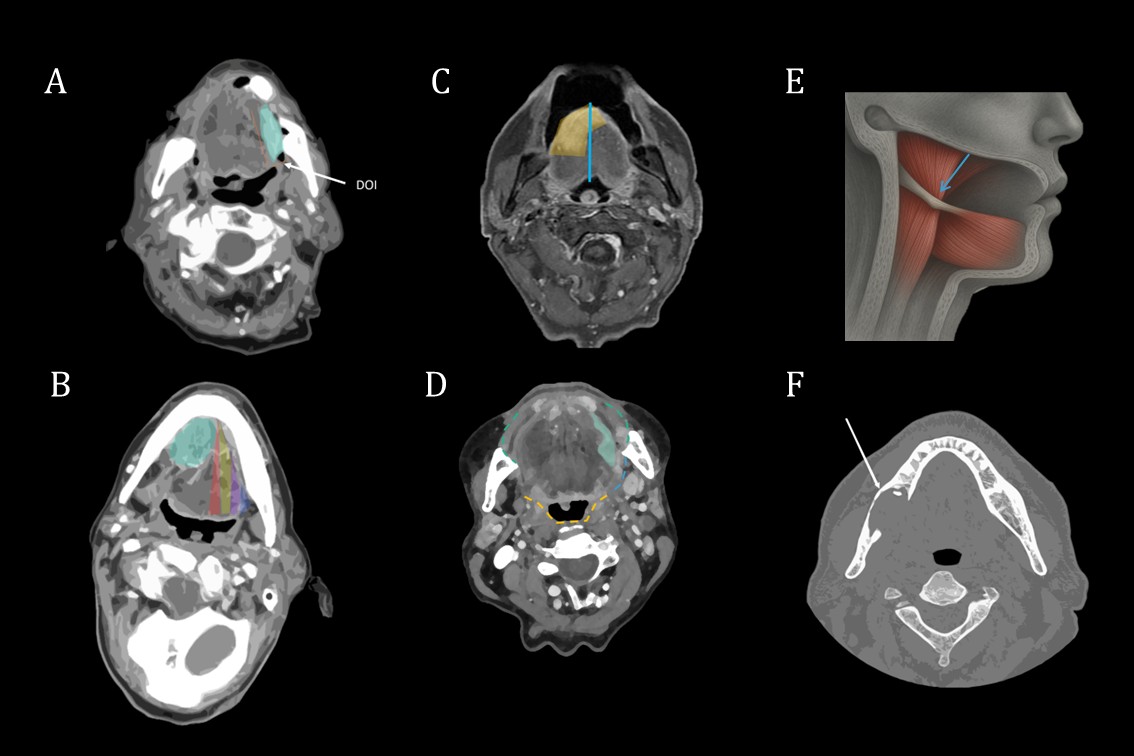


1. Axial section. Measurement of tumor depth of invasion (DOI).
2. Axial section. The genioglossus (red), hyoglossus (yellow), and styloglossus (purple) muscles are shown. In addition, involvement of the floor of the mouth, including the mylohyoid muscle (blue), must be assessed. The tumor is marked in green.
3. Axial section. The tumor is shown in orange, and a blue line indicates the midpoint.
4. Axial section. Tendinous complex located between the pterygoid hamulus cranially and the mandible caudally; this represents the insertion point of the buccinator muscles (green) and the superior pharyngeal constrictor muscles (yellow).
5. Simulated image. The arrow indicates the pterygomandibular raphe.
6. Axial section. The arrow indicates an area of bone erosion.


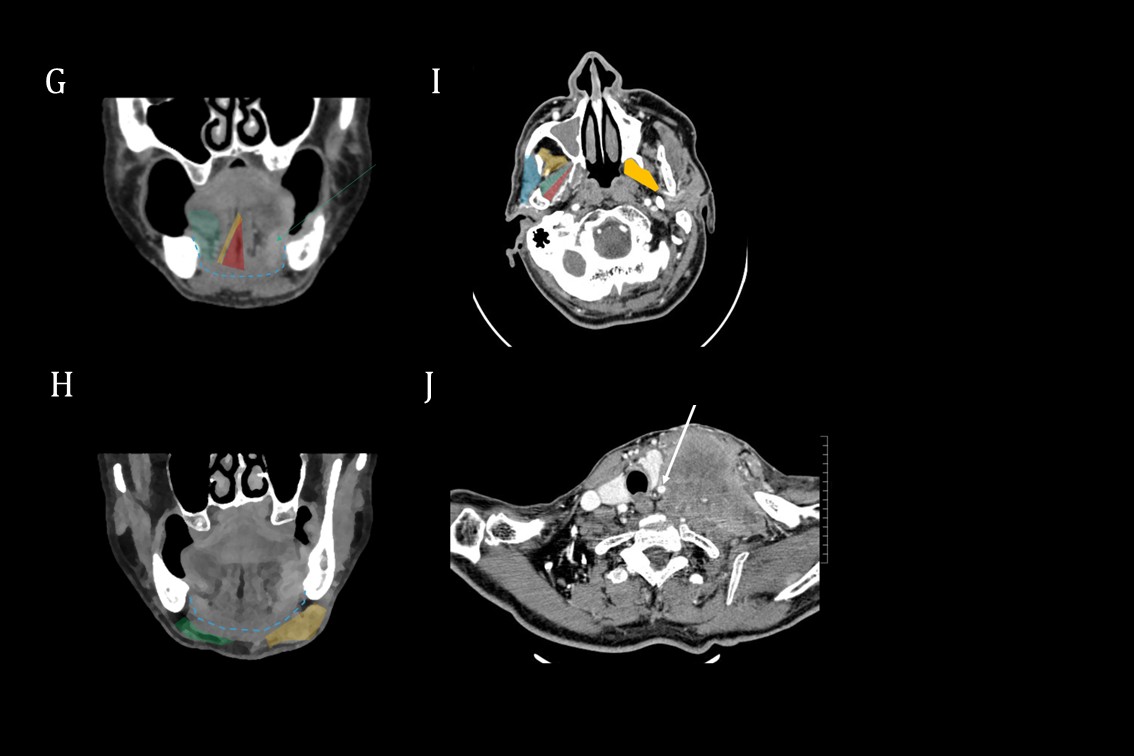


1. Axial section. The sublingual space is shown in green, the genioglossus muscle in red, and the hyoglossus muscle in yellow. The tumor is shown in gray and indicated by a white arrow.
2. Coronal section. A dashed blue line indicates the mylohyoid muscle; the space is shown in green and the tumor in yellow.
3. Axial section. Masticator space (orange); medial pterygoid muscle (red); lateral pterygoid muscle (green); masseter muscle (blue); temporalis muscle (yellow)**.**
4. Axial section. The arrow indicates the internal carotid artery compressed by an external tumor.
